# Periprostatic Nerve Block During Transperineal Prostate Biopsy Under General Anaesthesia: Protocol for a Multicentre Pilot Randomised Controlled Trial

**DOI:** 10.3390/cancers18081251

**Published:** 2026-04-15

**Authors:** Basil Razi, Cheryl Fung, George McClintock, Hadia Khanani, Nariman Ahmadi, James Symons, Henry H. Woo

**Affiliations:** 1 Department of Urology, Blacktown Mount Druitt Hospital, Sydney 2148, Australia; 2 Department of Uro-Oncology, Chris O’Brien Lifehouse, Camperdown, Sydney 2050, Australia; 3 Department of Urology, Sydney Adventist Hospital, Wahroonga, Sydney 2076, Australia; 4 Blacktown and Mount Druitt Clinical School, Western Sydney University, Sydney 2751, Australia

**Keywords:** anaesthesia, periprostatic nerve block, post-operative pain, prostate biopsy, prostate cancer, randomised controlled trial, visual analogue scale

## Abstract

**Background**: Transperineal prostate biopsy (TPPB) has become the preferred diagnostic approach due to superior apical sampling and a lower risk of post-biopsy sepsis, without compromising cancer detection. Although routinely performed under general anaesthesia in Australia, early post-operative pain remains common. Periprostatic nerve block (PPNB) improves tolerability when TPPB is performed under local anaesthesia, but its efficacy as an adjunct under general anaesthesia is unknown. This pilot trial evaluates whether PPNB reduces early post-operative pain and analgesic requirements following TPPB under general anaesthesia. **Clinical Trial Design and Timeframe**: A multicentre, double-blind, RCT conducted across three tertiary centres in Sydney, Australia. Men undergoing TPPB under general anaesthesia are randomised 1:1 to receive bilateral PPNB with lignocaine or placebo immediately prior to biopsy. **Endpoints**: The primary endpoint is post-operative pain measured using a 10-point visual analogue scale prior to discharge. Secondary endpoints include post-operative analgesic consumption, complication rates, procedure duration, and number of biopsy cores. **Data Sources and Statistical Analysis Plan**: Clinical and outcome data are collected prospectively. Between-group comparisons will be performed using independent-samples *t*-tests or Mann–Whitney U tests, as appropriate. Categorical outcomes will be analysed using χ^2^ or Fisher’s exact tests. Statistical significance is defined as *p* < 0.05. **Strengths and Limitations**: This multicentre, double-blind randomised design strengthens internal validity, although the modest sample size and reliance on early patient-reported pain may limit detection of smaller effects and longer-term outcomes.

## Simple Summary

Transperineal prostate biopsy is commonly used to diagnose prostate cancer and is usually performed under general anaesthesia only in Australia. Many men experience pain after the procedure while recovering. This study is examining whether an additional local anaesthetic injection around the prostate, known as a periprostatic nerve block, can reduce pain after biopsy. While this injection improves comfort when biopsies are performed under local anaesthesia, it is not known whether it provides benefit when the procedure is done under general anaesthesia. Men undergoing transperineal prostate biopsy will be randomly assigned to receive either the nerve block or a placebo injection before the biopsy. Neither the patient nor the treating team will know which injection is given. Pain levels, use of pain relief medication, and any complications will be assessed before discharge. The findings of this study will help determine whether adding a periprostatic nerve block improves recovery and patient comfort after prostate biopsy performed under general anaesthesia.

## 1. Introduction

Prostate biopsy remains the definitive diagnostic test for the diagnosis of prostate cancer. Two approaches are practised: transrectal and transperineal. The transperineal route has emerged as the preferred technique in many centres worldwide because it allows superior apical sampling and markedly reduces post-biopsy sepsis, without compromising cancer detection compared to a transrectal approach [1,2]. Despite these advantages, some studies have reported higher pain scores with the transperineal approach [3,4,5]. Patient-reported pain is influenced not only by the procedure itself, but also by pre-procedural expectations and anxiety [6], highlighting the need for strategies to optimise tolerability.

In Australia, transperineal prostate biopsy (TPPB) is almost exclusively performed under general anaesthesia, and local anaesthesia is not routinely employed. Nevertheless, 30–50% of patients experience post-procedural pain, and approximately 10% reporting severe pain in the immediate post-operative period [7,8]. To mitigate procedural pain, various local and regional anaesthesia techniques have been reported in the current literature. In several international centres TPPB is increasingly performed under local anaesthesia, in which periprostatic nerve blocks (PPNB) form an integral component of the anaesthetic technique to optimise patient comfort and procedural tolerability [9,10].

The PPNB, originally developed for transrectal biopsy, targets the neurovascular bundle between the prostate and seminal vesicles. It can be performed quickly and safely, under direct ultrasound guidance, and carries minimal additional morbidity [11,12]. Multiple studies have shown that incorporating a PPNB during TPPB improves pain scores, reduces procedural discomfort and overall procedure tolerability [6,9,13].

There are no randomised controlled trials evaluating the role of PPNB in the setting of TPPB performed under general anaesthesia. It is biologically plausible that a supplementary periprostatic block could attenuate nociceptive input during biopsy, thereby reducing early post-operative pain, limiting the need for analgesics, and improving the overall recovery experience. Therefore, this multicentre randomised trial has been designed to test this hypothesis and to determine whether PPNB should be incorporated into routine practice for patients undergoing TPPB under general anaesthesia.

## 2. Patient and Methods

### 2.1. Study Design

This is a prospective, multicentre, randomised controlled trial (RCT) designed to evaluate the efficacy of PPNB in men undergoing TPPB under general anaesthesia. Participants will be recruited from three centres in Sydney, Australia: Chris O’Brien Lifehouse, Sydney Adventist Hospital, and Blacktown District Hospital.

The primary objective of this trial is to determine whether the addition of a periprostatic nerve block reduces post-operative pain, measured using a 10-point visual analogue scale (VAS) prior to discharge, in men undergoing transperineal prostate biopsy under general anaesthesia.

The secondary objectives are to evaluate whether the use of a periprostatic nerve block is associated with differences in complication rates compared to placebo, and to assess whether it reduces the requirement for post-operative analgesia.

This study has received ethical approval from the Sydney Local Health District Human Research Ethics Committee (2020/ETH03024) and has been registered with the Australian New Zealand Clinical Trials Registry (ACTRN12621001143819). All procedures will be performed in accordance with the National Statement on Ethical Conduct in Human Research (2018), Good Clinical Practice guidelines, and the principles of the Declaration of Helsinki. Trial conduct will be overseen by the principal investigator at each site, with central coordination by the chief investigator and support from a study coordinator.

At the study conclusion, a formal data analysis will be undertaken to obtain the result of this study. A schematic SPIRIT format of the study design is outlined in Table 1.

### 2.2. Eligibility Criteria

Inclusion criteria

- Adults, age ≥ 18 years
- Male patients scheduled for TPPB under general anaesthesia
- Ability to provide informed consent

Exclusion criteria

- Inability to provide informed consent or is unwilling to participate in this study
- Known allergy or prior anaphylaxis to local anaesthetic agents
- Received locoregional nerve block/anaesthesia within 24 h prior to biopsy
- Unable to communicate, read or write in English to a level where they are able to understand the study and consent to inclusion

### 2.3. Patient Recruitment and Screening

Potential participants will be identified through private rooms or urology outpatient clinics at the time of their initial consultations. Patients scheduled to undergo TPPB for the investigation of suspected prostate cancer will be screening against the eligibility criteria by the treating urologist or delegated clinical research member.

All eligible patients will be provided with Participant Information and Consent Forms at their initial consultations. Patients will be encouraged to review these documents at home and are not required to make an immediate decision. Informed consent may be provided on the day of the biopsy or at any time prior to the scheduled procedure. During this period, prospective participants may contact the research team by phone or email to address any questions or concerns.

Screening will include a review of medical history, clinical details, and allergy status. Eligible and consenting patients will be assigned a unique study identification number, which will be recorded on all study documentation and electronic medical records (EMR). Enrolled participants will then proceed to randomisation on the day of biopsy.

Participants may withdraw from the study at any time without prejudice to their ongoing care. Withdrawal can be communicated verbally by speaking directly with a member of the research team, or via telephone or email. While participants will be invited to complete a withdrawal form for record-keeping purposes, this is not mandatory; verbal notification will be considered sufficient.

### 2.4. Randomisation

After informed consent, participants will be randomised in a 1:1 ratio to receive either:

- Intervention group: PPNB administered with 20 mL 1% lignocaine with 1:100,000 adrenaline (approved for use by the Therapeutic Goods Administration and listed on the Australian register of Therapeutic Goods (ARTG). The ARTG is a register of therapeutic goods that can be lawfully supplied in Australia), injected under ultrasound guidance immediately prior to biopsy
- Control group: placebo injection with 20 mL sterile 0.9% normal saline, injected under ultrasound guidance immediately prior to biopsy

The randomisation sequence will be computer generated using variable block sizes (2, 4 and 6); stratification will be by site. The allocation sequence will be prepared off-site by a trained study secretary, who will sequentially number and seal opaque envelopes. A box containing the required number of envelopes for use at each site will be made.

On the day of biopsy, a trained scout nurse will remove a sealed envelope from the box outside the operating theatre. The envelope will be opened, and the allocated injectate (PPNB or placebo) will be prepared in an unlabelled syringe of identical appearance. The scout nurse, who is not involved in outcome collection, will remain the only unblinded team member. Patients, surgeons, anaesthetists, and research staff collecting outcome data and performing analysis will remain blinded to allocation.

To reduce error, each envelope box will also contain a randomisation record, to be completed by the scout nurse at each location (Supplementary File S1). This document includes patient details, allocation, and solution preparation instructions, and will remain secured within the envelope box. A flowchart of instructions will also be affixed to each box to support adherence to protocol.

### 2.5. Intervention

All enrolled participants will undergo TPPB under general anaesthesia and will be randomised as outlined earlier. Immediately prior to biopsy, patients will undergo a periprostatic nerve block (intervention arm) or a placebo injection (control arm).

The PPNB will be performed under ultrasound guidance using a spinal needle advanced transperineally to the space between the prostate and seminal vesicles, targeting the neurovascular bundles bilaterally. A total of 20 mL of 1% lignocaine with adrenaline will be administered (10 mL on each side), with local anaesthetic visualised during injection to confirm placement and minimise risk of extravasation.

The placebo injection will consist of 20 mL sterile 0.9% normal saline, delivered using the same technique. Both active and placebo injectates will be prepared by the unblinded scout nurse in unlabelled syringes of identical appearance to maintain blinding.

Following injection, transperineal prostate biopsy will proceed in the standard manner under general anaesthesia. The biopsy template and number of cores obtained will be determined by clinical factors.

Following the procedure, and prior to discharge, each patient will complete a VAS pain score form (Supplementary File S2). A clinical research team member will collect this form and record any additional analgesia administered post-operatively, on the same form.

Risks and Safety

PPNB is a well established and low risk adjunct to prostate biopsy. The most common adverse effects are pain and bruising at the injection site. Uncommon (1 in 1000) risks include nerve block failure, temporary numbness, allergic reaction to lignocaine, and iatrogenic injury to blood vessels, muscles or nerves. Very rare (1 in 10,000) complications include local anaesthetic systemic toxicity, seizures, or anaphylaxis. All injectates used in this study are approved for clinical use by the Therapeutic Goods Administration.

Patients may experience side effects or serious adverse events related to their TPPB, which will be managed as per clinical protocols and treated accordingly. Investigators will make records of any side effects or serious adverse events, as they are relevant to the scope of the study. This is in line with the NHMRC Safety Reporting and Monitoring in Clinical Trials (2016).

### 2.6. Endpoints

Primary endpoint

- Post-operative pain: measured using a 10-point visual analogue scale completed by the participant prior to discharge following TPPB. This is approximately 3–4 h post general anaesthesia, reflecting standard hospital protocols, resulting in a consistent and comparable timeframe across study participants.

Secondary endpoints

- Post-operative analgesia requirements, recorded as type, route, and dose, converted to oral morphine equivalent
- Rate and severity of complications arising from use of PPNB
- Procedure duration and number of biopsy cores obtained

### 2.7. Sample Size

The primary outcome of interest is the between-group difference in the post-procedure VAS scores between the intervention (PPNB) and placebo injection. A meta-analysis of transrectal prostate biopsies demonstrated that PPNB was associated with a considerable reduction in post-procedural pain, with a pooled standardised mean difference of −1.27 (95% CI −1.72 to −0.82) compared with placebo [14]. Although this suggests a large effect size, the analysis also reported significant heterogeneity (I^2^ = 92%), reflecting variability across trials. A separate review of acute pain interventions reported that the minimum clinically important difference ranged from 0.4 to 4.0, with a median of 1.7 on a 10-point VAS [15].

On this basis, the sample size calculation assumed a between-group difference of 1.5 on the VAS, with a pooled standard deviation of 1.75, corresponding to a large effect size (Cohen’s *d* = 0.86). Using a two-sided α of 0.05 and 80% power, the required sample size is 22 patients per group. Allowing for dropout, a total of 50 patients will be recruited (25 per group). The sample size calculation was performed using G*Power v3.1.9.6 [16].

### 2.8. Data Collection and Management

Baseline demographic and clinical data will be collected from medical records, including age, prostate volume (from pre-operative imaging), and previous prostate biopsies (number and type). Intra-operative data collected include number of cores and duration of procedure.

The primary outcome, post-procedure pain, will be assessed using a 10-point VAS score completed by participants prior to discharge. Secondary outcomes, including type and dose of post-operative analgesia, will be documented by the clinical research team and converted to oral morphine equivalents for analysis. Complications will be recorded prospectively during the admission and retrospectively through review of electronic medical records, with severity graded according to the Clavien–Dindo classification. This is summarised in Table 2.

All outcome data will be entered prospectively (coded) into a secure database hosted by Western Sydney Local Health District. Each participant will be assigned a unique study identification number, which will be used on all study forms and electronic entries. Identifiable information will be stored separately from coded data and accessible only to the principal investigators at each site. Access to the data is password protected and limited to only the named research investigators. Data will be retained for 15 years following study completion in accordance with local NHMRC Code for The Australian Code for the Responsible Conduct of Research. At the end of the retention period, electronic records will be permanently deleted and paper records securely destroyed.

### 2.9. Statistical Analysis Plan

Descriptive statistics will be presented on baseline characteristics of the two groups. Continuous variables reported as mean (standard deviation) or median (interquartile range), while categorical variables will be reported as frequencies and percentages.

The primary outcome, post-operative pain measured by VAS, will be compared between groups using an independent-samples *t*-test if normally distributed, or a Mann–Whitney U test if non-normally distributed.

Secondary outcomes will be analysed as follows:

- Post-operative analgesia requirements (oral morphine equivalents) will be compared using *t*-tests or Mann–Whitney U tests, depending on data distribution.
- Complication rates will be compared between groups using χ^2^ tests or Fisher’s exact tests where appropriate.
- Procedure duration and number of biopsy cores will be analysed using *t*-tests or non-parametric equivalents.

All statistical tests will be two-sided, with a significance level set at *p* < 0.05. Effect sizes with 95% confidence intervals will be reported for primary and secondary outcomes. Missing data will be handled using complete case analysis. Statistical analysis will be performed using SPSS (Version 29, Armonk, NY, USA).

In the event of significant missing data, a sensitivity analysis will be performed to assess the robustness of these findings.

## 3. Discussion

Analgesia in prostate biopsy is an underappreciated but important clinical aspect affecting patients, influencing pre-procedural anxiety and post-procedural discomfort and analgesic needs, even if performed under general anaesthesia. While TPPB has increasingly replaced the transrectal approach due to better prostate sampling and reduced risk of sepsis, which is reflected in the European Association of Urology Guidelines’ strong recommendation [17], it is more painful than the traditional transrectal approach. In the contemporary era of active surveillance, where men may undergo repeat biopsies, optimisation of post-procedural discomfort is important not only to reduce immediate morbidity but also to support long-term adherence to surveillance protocols.

Analgesic strategies in prostate biopsy historically focus on transrectal approach, where PPNB has strong evidence from systematic reviews and meta-analyses supporting its efficacy, with or without adjuncts such as intrarectal local analgesia, in reducing pain compared to placebo or no injection [18,19]. A recent RCT comparing perineal nerve block to PPNB in TPPB under local anaesthesia showed that perineal nerve block had lower pain scores during and at 1 h post-procedure [20]. This suggests that the optimal analgesia for TPPB differs from the transrectal approach.

There is also important heterogeneity in real-world TPPB practice that warrants consideration when interpreting this study. Variability exists not only in biopsy technique, but also in operator experience and training pathways. In a prospective study of naïve trainees, different technical approaches were associated with differences in procedural efficiency and cancer detection in specific subgroups, highlighting the influence of operator learning curves and technique selection on outcomes [21]. Furthermore, contemporary international survey data demonstrates substantial variability in trainee exposure to TPPB, with fewer than half of urology trainees reporting hands-on experience and marked differences between countries and institutions [22]. This heterogeneity extends to procedural independence, with proficiency closely linked to procedural volume.

These findings underscore that biopsy outcomes, such as patient-reported pain, may be influenced by operator-dependent factors such as experience, technical familiarity, and institutional practice patterns. Some degree of inter-operator and inter-centre variability is unavoidable and reflects pragmatic, real-world practice. In Australia, however, TPPB under general anaesthesia is the common and preferred approach. Thus, both trainees and consultant urologists in this study would have substantial exposure to and familiarity with the procedure, reducing the degree of operator variability compared to international settings where TPPB adoption remains inconsistent. Importantly, this enhances the external validity of the findings, although it may introduce additional variability in patient-reported outcomes.

Nevertheless, to date there is no high-quality evidence from randomised, double-blind, controlled trials examining PPNB (versus placebo) during TPPB under general anaesthesia. This multicentre trial is designed to address this evidence gap by providing blinded, controlled data on the effect of PPNB on early post-operative pain, analgesic use, and safety outcomes. However, the biggest challenge of this trial is that patient-reported pain is inherently subjective and may be influenced by individual pain thresholds, expectations, and peri-operative anxiety. Heterogeneity may also be introduced by the differences between centres in small variations in biopsy techniques.

### 3.1. Strengths

This multicentre, double-blinded, randomised controlled design is a key methodological strength, minimising selection and detection bias. Stratified randomisation by site also reduces site-specific confounding. The use of concealed allocations preserves the blinding of patients, surgeons, anaesthetists and outcome assessors. The protocolised PPNB technique and standardised outcome measurement strengthens the study’s reproducibility. Objective endpoints are achieved via standard conversion to oral morphine equivalents. The pragmatic inclusion of three tertiary centres also further strengthens the external validity within Australia.

### 3.2. Limitations

However, there are some limitations inherent to this study. The modest sample size, powered to detect a clinical difference in early post-operative pain, may limit ability to detect smaller differences in secondary outcomes. Pain is inherently subjective and influenced by patient pain thresholds; in addition, peri-operative anxiety and recovery room factors may introduce variability. Variations in biopsy template, number of cores and subtle inter-operator technique differences across centres may introduce heterogeneity, although this reflects real-world practice.

## 4. Conclusions

Regardless of direction, the results of this trial will offer robust, clinically relevant evidence for practitioners and may inform future guidelines on analgesic practice in TPPB. If PPNB demonstrates meaningful benefit, it could be considered as a routine adjunct. Conversely, a null finding would suggest that additional injections are unnecessary, streamlining procedural workflows. In either case, this trial may encourage further exploration of block strategies (e.g., perineal nerve block, pudendal block) and analgesia optimisation in prostate biopsy practice.

## Figures and Tables

**Table 1:** SPIRIT format of the study design.

Study Activities	Initial Consultation	Visit 1 (Day of Biopsy)
Informed consent	✓	
Inclusion/Exclusion Criteria	✓	
Physical examination	✓	
Baseline data collection and Randomisation		✓
Transperineal prostate biopsy ± periprostatic nerve block		✓
Post-operative pain questionnaire		✓

**Table 2:** Data collection per patient.

Domain	Variable	Definition	Source
Baseline	Age	Years at biopsy	Medical record
	PSA	Most recent PSA	Medical record
	Prostate volume	mL on MRI	Imaging
	Prior biopsy	Number and type	Medical record
Intra-operative	Procedure duration	Minutes	Operative note
	Cores taken	Number of cores	Operative note
Primary outcome	Post-op pain	VAS (0–10)	Patient survey
Secondary outcome	Analgesia use	Oral morphine equivalent (mg)	Medication chart
	Complications	Clavien–Dindo classification	Medical record review

## Data Availability

The original contributions presented in this study are included in the article/Supplementary Material. Further inquiries can be directed to the corresponding author.

## Supplementary Materials

The following supporting information can be downloaded at: https://www.mdpi.com/article/10.3390/cancers18081251/s1, File S1: Randomisation Record; File S2: Data Collection Vas.
