# Supplementary material for: Periprostatic Nerve Block During Transperineal Prostate Biopsy Under General Anaesthesia: Protocol for a Multicentre Pilot Randomised Controlled Trial
Source: Cancers (Basel). 2026 Apr 15;18(8):1251. doi: 10.3390/cancers18081251 (PMC13114603; doi:10.3390/cancers18081251)
Supplement: Supplementary file 1 [file cancers-18-01251-s001.zip › cancers-4221410-supplementary.pdf]

Supplementary File S1 – Randomisation Record

RANDOMISED TRIAL OF PERIPROSTATIC NERVE BLOCK IN TRANSPERINEAL PROSTATE BIOPSY UNDER GENERAL ANAESTHESIA

RANDOMISATION RECORD – PROF. HENRY WOO

A randomised trial of periprostatic nerve block in transperineal prostate biopsy under general anaesthesia

Thank you for assisting with this clinical trial. We are aiming to determine if local anaesthetic as a nerve block during transperineal prostate biopsy reduces post-operative pain.

You have been asked to complete this task because the patient, surgeon and anaesthetist should not know which patients are being administered local anaesthetic and which are being given a placebo.

Your job today is to randomise the patient and to make up either the local anaesthetic solution or the placebo solution.

A flow diagram of a step-by-step process is attached on the box for ease of direction.

Things to remember before you begin:

- Make sure the name of the surgeon on the box is the same on top of this document and is the one operating today.
- Do not open the envelope until you have the patient details recorded.
- Do not share the assignment with anyone. In the event of an emergency or reaction, you may reveal the assignment.
- Do not recruit more than twenty-five patients.

For any issues contact: Professor Henry Woo, henry.woo@health.nsw.gov.au

Randomisation Record- to be filled out by nurse only

DATE	Envelope	Patient NAME and MRN
	1	
	2	
	3	
	4	
	5	
	6	
	7	
	8	
	9	
	10	
	11	
	12	
	13	
	14	
	15	
	16	
	17	
	18	
	19	
	20	
	21	
	22	
	23	
	24	
	25	

Supplementary File S2 – Data Collection Vas

RANDOMISED TRIAL OF PERIPROSTATIC NERVE BLOCK IN TRANSPERINEAL PROSTATE BIOPSY UNDER GENERAL ANAESTHESIA

DATA COLLECTION – PROF. HENRY WOO

**Ethics Protocol**	2020/ETH03024
**Principal Investigator**	Prof. Henry Woo
**Institution**	Department of Urology<br>Blacktown Hospital<br>18 Blacktown Road, Blacktown<br>NSW, 2148, Australia

Investigator to complete (or patient sticker)

**Study Enrolment Number (Study ID)**	
**Patient Name (First, Last)**	
**MRN**	
**DOB**	
**Date of Biopsy**	
**Consultant**	

Patient to Complete:

Please mark on the line below your WORST level of pain after the procedure today:

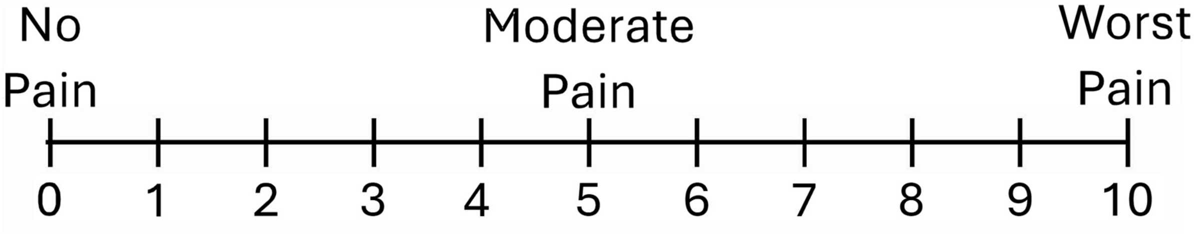

Investigator to complete:

Please fill out any additional analgesia used post-operatively. Leave blank if none used.

**Analgesic**	**Doses**	**Route**	**Total Dose**

Were there any complications prior to discharge? ☐ Yes ☐ No

Details:

____

____

Did the patient withdraw from the study? ☐ Yes ☐ No

If yes, reason (s) for withdrawal (if any):

____

____
